# Developing Sampling Plans for the Verification of the Effectiveness of Technical Measures: An Erosion and Sediment Control Case Study to Protect Water Quality

**DOI:** 10.1007/s00267-026-02580-4

**Published:** 2026-09-03

**Authors:** Roland Cormier, Manon Mallet, Gabriel Goguen

**Affiliations:** 1 National Centre for Effectiveness Science, Fisheries and Oceans Canada, 343 Université Avenue, Moncton, NB E1C 5K4 Canada; 2 Gulf Fisheries Centre, Fisheries and Oceans Canada, 343 Université Avenue, Moncton, NB E1C 5K4 Canada

## Abstract

We demonstrate the use of acceptance sampling by attribute to verify that technical measures implemented at construction worksites meet the requirements of environmental regulations, standards and codes of practice. In contrast to environmental monitoring of broader spatial and temporal effects, acceptance sampling plans are used to verify the effectiveness of such measures at the time of the verification during construction activities. Applying power statistics used in acceptance sampling, Type II Error (β) is considered as the environment’s risk to achieving environmental protection policies while Type I Error (α) is considered as the proponent’s risk for the person having to implement such measures. Using a case study, we demonstrate the development of acceptance sampling plans by attribute for erosion and sediment control. Working from a hypothetical policy for the environment’s risk and the proponent’s risk, we also underline the importance of sample sizes to make the correct decisions when verifying if technical measures are functioning as intended based on a policy for acceptable levels of non-conformity. The report provides an overview of the probability mathematics used in acceptance sampling plans that include the calculation of the acceptance number as a decision rule for a given sample size and the calculations of the environment’s risk and the proponent’s risk.

## Introduction

Technical measures are the operational implementation of ecosystem-based management approaches to achieve environmental policy objectives (Cormier and Elliott, 2022a; Elliott et al. 2025b). Technical measures can be implemented through regulatory and non-regulatory instruments as part of environmental legislation. These provide the technical details needed to carry into effect the objectives of legislation and within an environmental policy context (Cormier et al. 2017). As permitting conditions for a worksite, technical measures specify the physical controls including operation, inspection, and maintenance procedures to prevent and mitigate potential spatial and temporal impacts generated from worksite activities (Cormier et al. 2022b). There are many technical measures used for the protection of the environment, for example, the guidelines for the protection of aquatic life (Canadian Council of Ministers of the Environment 2022) including industry standards and codes of practice (Province of British Columbia 2022; Province of New Brunswick 2012; Toronto and Region Conservation Authority 2019; Canadian Standards Association Group 2020). In addition, environmental protection policies also define the intended functions of technical measures such as avoidance and mitigation measures for the protection of fish and fish habitat (Fisheries and Oceans Canada 2019a, 2024, 2025a). The practical implementation of technical measures at a worksite is through physical controls that operate as a system of controls (Goguen et al. 2025; Cormier and Elliott, 2022a).

Many environmental monitoring approaches and tools are used to assess the impacts of human activities and their effects on the environment within the context of environmental impact assessments and the state of the environment (Papadopoulou et al. 2025). The design of such monitoring plans needs to consider the duration and variability of the indicators being monitored to detect trends over time (Portt et al. 2006; Lamothe and Drake 2025). The design most often requires before-after-control-impact monitoring to compare the control site and the impact sites before a development project starts and to continue after its completion (Fisheries and Oceans Canada 2019b; Pou-Rovira et al. 2022; Tabor et al. 2022). Monitoring provides the basis to determine if environmental management and development strategies are performing as intended to achieve environmental protection policy objectives (Elliott et al. 2025a).

In Canada, there are multiple technical measures that are required for worksite construction activities that occur near or in water (Province of New Brunswick 2012; Fisheries and Oceans Canada 2025a). Implemented through standards and codes of practices (Fisheries and Oceans Canada 2026a, 2026b), the required technical measures are most often listed as conditions of authorizations or permits as part of an approval process that include a review of the construction activities (Fisheries and Oceans Canada 2025b). Once an authorization or a permit is issued, the required technical measures are installed during worksite preparations. Throughout the construction activities, the technical measures further specify requirements for inspections and maintenance. Once the construction activities are completed, the worksite is reinstated to natural processes which may include additional restoration technical measures of the watercourse and riparian zone. During the construction phase, some technical measures are used for short periods of time compared to others. For example, erosion and sediment control would be needed for the entire duration of the construction phase whereas the capture and relocation of fish when a worksite is dewatered may only take a few days to a week as part of the construction operation.

While technical measures are being used, there is a need to verify that the configuration and installation of these are meeting their specified expected outcomes effectively. In contrast to environmental monitoring discussed above, a verification must confirm effectiveness of the technical measures in the immediate term to make the timely corrections needed while the construction activities are taking place. Here, we propose the use of acceptance sampling by attribute to verify the effectiveness of technical measures. Acceptance sampling by attribute is widely used in quality assurance of products and in performance evaluation of systems of controls to determine if a product or process is meeting regulatory or non-regulatory requirements (Göb and Baillie 2012). Based on the ISO 2859 standard for sampling procedures for inspection by attributes (International Organization for Standardization 2017), acceptance sampling is used to ensure that a product, a system, or a process meet specified quality levels for a given attribute at the time of the inspection. ISO 2859 recognizes the need to take into account the human and financial resources to conduct such inspection in terms of the sample sizes needed to make such decisions. ISO 2859 also stipulates that ad hoc sampling, or random checks may not provide the needed evidence to demonstrate the conformity leading to higher risks to the consumer.

For the development of an acceptance sampling plan based on ISO 2859, the attribute defines what is conformity during an inspection. A non-conformity is a departure from the quality characteristics defined by the conformity attribute. In addition, the level of non-conformities for a satisfactory process average is used to develop acceptance sampling policies for acceptable and rejectable quality levels (ISO 2859) (International Organization for Standardization 2017). Based on the conformity attribute and satisfactory process average, a sampling plan and decision rules can be established to address the risk of the producer for producing the quality and the consumer expecting the quality. Using this approach in an environmental context, the conformity attribute needs to be framed by the expected outcome of the technical measures (Cormier et al. 2022b, Reynolds et al. 2016). Here, the system performance quality level of these technical measures, considered as equivalent to satisfactory process average, is subsequently needed to develop the sampling policies for acceptable and rejectable quality. In addition, the proponent and the environment are considered as the producer and consumer, respectively.

Erosion and sediment control are used internationally such as those of the Erosion Control Association Design Standards (International Erosion Control Association 2026). We demonstrate the development of an acceptance sampling plan that could be used to verify the effectiveness of erosion and sediment control used during the replacement of two road stream crossings as a case study. Given the Canadian regulatory context of this paper, the national standard for erosion and sediment control is used for the case study (Canadian Standards Association Group 2018). The conformity attribute is based on the water quality management benchmark of the CCME guidelines for total suspended sediment (Canadian Council of Ministers of the Environment 2002). The system performance quality level for such technical measures is based on the more detailed and data rich reliability study from Goguen et al. (2025). We also introduce the use of Type I Error to quantify the risk to the proponent and Type II Error to quantify the risk to the environment based on an analysis of the statistical power of the sampling plan.

## Effectiveness Verification vs. Reliability Analysis

**Fig. 1:**
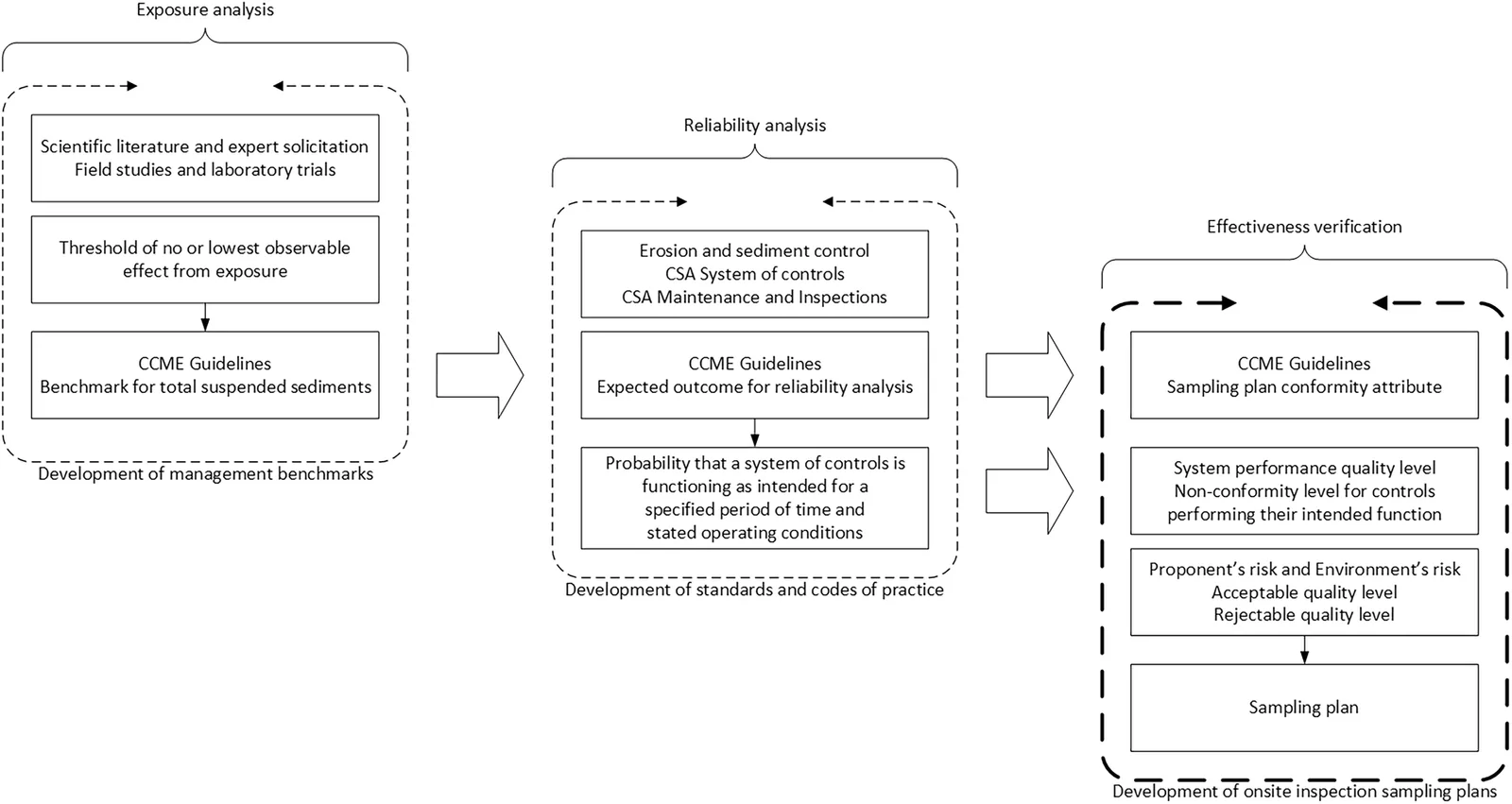
Key elements for developing an effectiveness verification sampling plan

Effectiveness verification is used to verify that the configuration of a system of controls implemented at a worksite is performing its intended function at the time of an onsite inspection. Because of the regulatory implications of conformity requirements when using acceptance sampling plans, the development of an effectiveness verification sampling plan discussed here is based on ISO 2859 (International Organization for Standardization 2017). Several steps are required before developing an effectiveness verification sampling plan (Fig. 1). The development of such a sampling plan requires metrics for a conformity attribute and a satisfactory process average for the type of process to be inspected. Adapted to an environmental management context, a management benchmark is needed for the conformity attribute of the effectiveness verification sampling plan. Such benchmarks are established through exposure analysis informed by advisory processes that involve the review of scientific literature and expert solicitation to establish a threshold for “no or lowest observable effects” (International Electrotechnical Commission 2019). In Canada, a similar approach is used to establish management benchmarks for the Canadian Water Quality Guidelines for the Protection of Aquatic Life (Canadian Council of Ministers of the Environment 2007). These management benchmarks are subsequently used to conduct a reliability analysis to develop standards and codes of practice for technical measures that are expected to function as intended for a specified period of time and stated operating conditions. For effectiveness verification, the benchmark becomes the conformity attribute, and the reliability of the standards and codes of practice become the system performance quality level that are used to develop onsite inspection sampling plans.

Given that the case study is erosion and sediment control, the benchmark established by the CCME guidelines for turbidity is used as the conformity attribute (Box 1) (Fig. 1). Since erosion and sediment control involves the configuration of a system of physical controls, such a system of controls would be considered effective when maximum increases of 8 Nephelometric Turbidity Units (NTU) from background levels occur for short-term periods.

Although reliability analysis and effectiveness verification may appear similar, the purpose of a reliability analysis is to structure the assessment of system performance for the development of standards and codes of practice for specific technical measures to be used across a broad range of project types and worksite characteristics. In contrast, the purpose of effectiveness verification sampling plans is to confirm in a timely manner that the application of the standards and codes of practice are meeting the requirements when used in practice at a worksite. It is noted that a reliability analysis requires large amounts of data collected for the entire duration of the project while effectiveness verification requires less data collected during an onsite inspection given the smaller sample size of an acceptance sampling plan.

**Fig. 2:**
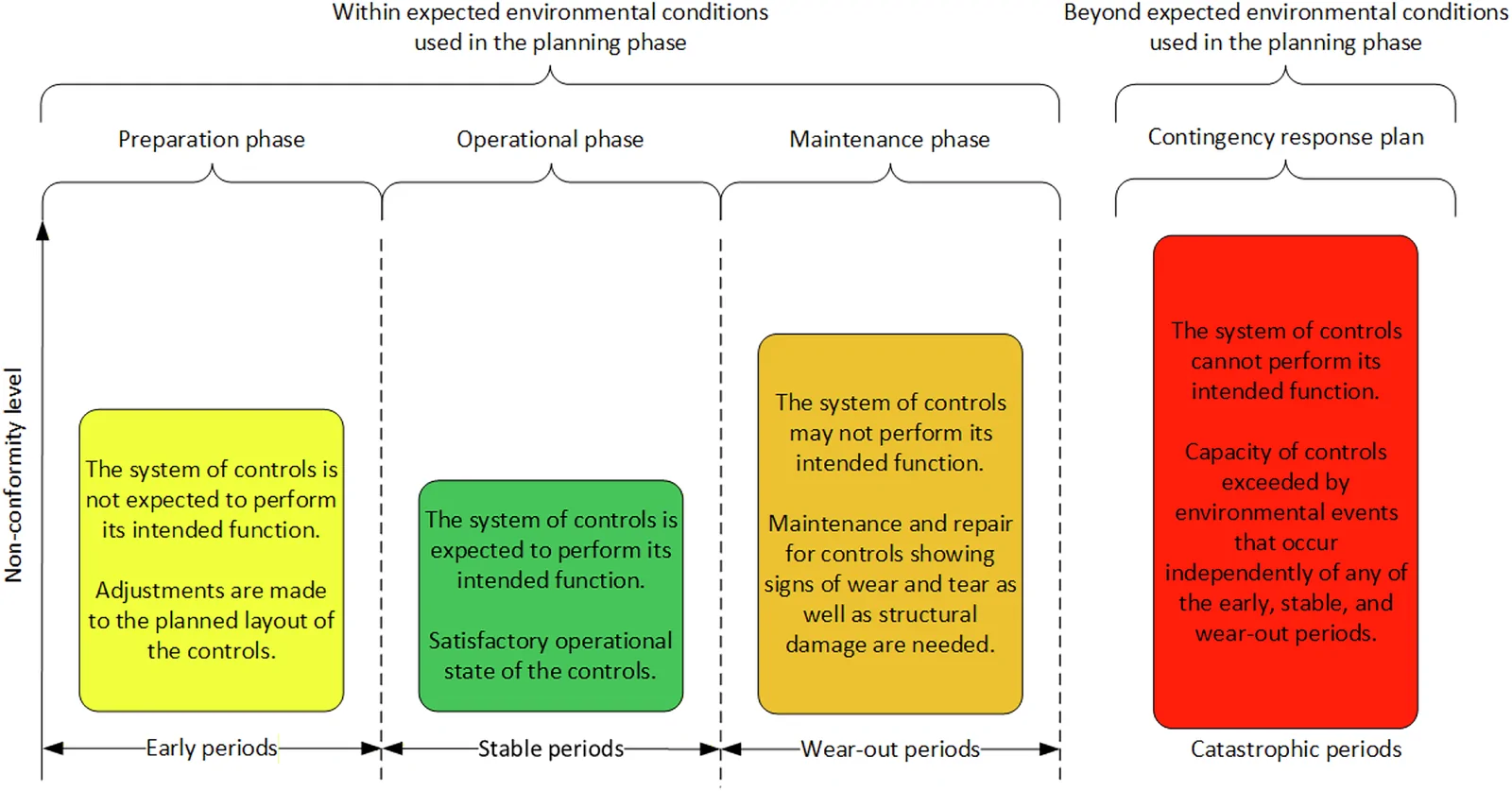
Bathtub Curve adapted to environmental technical measures (adapted from Goguen et al. 2025)

Reliability analysis techniques are commonly used to make improvements to the design and the reliability of controls or a system of controls to ensure that these function as expected in a cost-effective manner (Breneman et al. 2022; O’Connor and Kleyner 2025). Reliability data are used to calculate the probabilities when a system is functioning as intended in addition to when they break down and fail. Based on the Bathtub curve (Fig. 2) (Center for Chemical Process Safety 2015; Tobias and Trindade 2012), the design and implementation of a system of controls is divided into three reliability periods. The early periods represent the period when the design is being adjusted while the system of controls is being installed. This is followed by the stable period which is the period that the system of controls is expected to perform its intended function. Ultimately, the controls within the system wear-out from use and require repairs or replacement in order to return the system of controls to a satisfactory performance. Given here that the system of controls is exposed to stochastic environmental events, the catastrophic periods are added to the analysis for situations where external factors exceed the stated operating conditions identified in planning that could lead to a failure of the system of controls. After such a catastrophic event, extensive repair work is often needed to restore the system to a satisfactory performance. The reliability of a system of controls is expressed in terms of the probability of failure during the stable period which is expected to occur at a constant rate and at a lower rate than the other periods.

Box 1 Turbidity Benchmark for Conformity Attribute (Canadian Council of Ministers of the Environment 2002) Purpose

• For the purposes of this fact sheet, guidelines for total particulate matter in freshwater, estuarine, and marine environments will be derived for suspended sediments, turbidity, deposited bedload sediments, and streambed substrate.

Turbidity

• Turbidity is a measure of the lack of clarity or transparency of water caused by biotic and abiotic suspended or dissolved substances. The higher the concentration of these substances in water, the more turbid the water becomes. Technically, when passing light through a water sample, turbidity is an expression of the optical properties of substances that causes light to be scattered and absorbed rather than transmitted in straight lines through the sample (Wetzel 1975). The most reliable method for determining turbidity is nephelometry (light scattering by suspended particles), which is measured by means of a turbidity meter giving nephelometric turbidity units (NTUs). Environmental samples vary within the normal range of 1 to 1000 NTUs (Chapman 1992).

Guidelines

- In clear stream systems, small, induced exceedances in suspended sediment concentration above a 25 mg⋅L^-1^ change from background levels for short-term exposure (e.g., 24 h) are likely to cause behavioral and low sublethal effects on fish, all of which are reversible.
  Conformity attribute
- Turbidity for clear flow: Maximum increase of 8 NTUs from background levels for a short-term exposure (e.g., 24 h period). Maximum average increase of 2 NTUs from background levels for a longer term exposure (e.g., 30-d period).

## Operational Context of Construction Worksite with Application to a Case Study

**Fig. 3:**
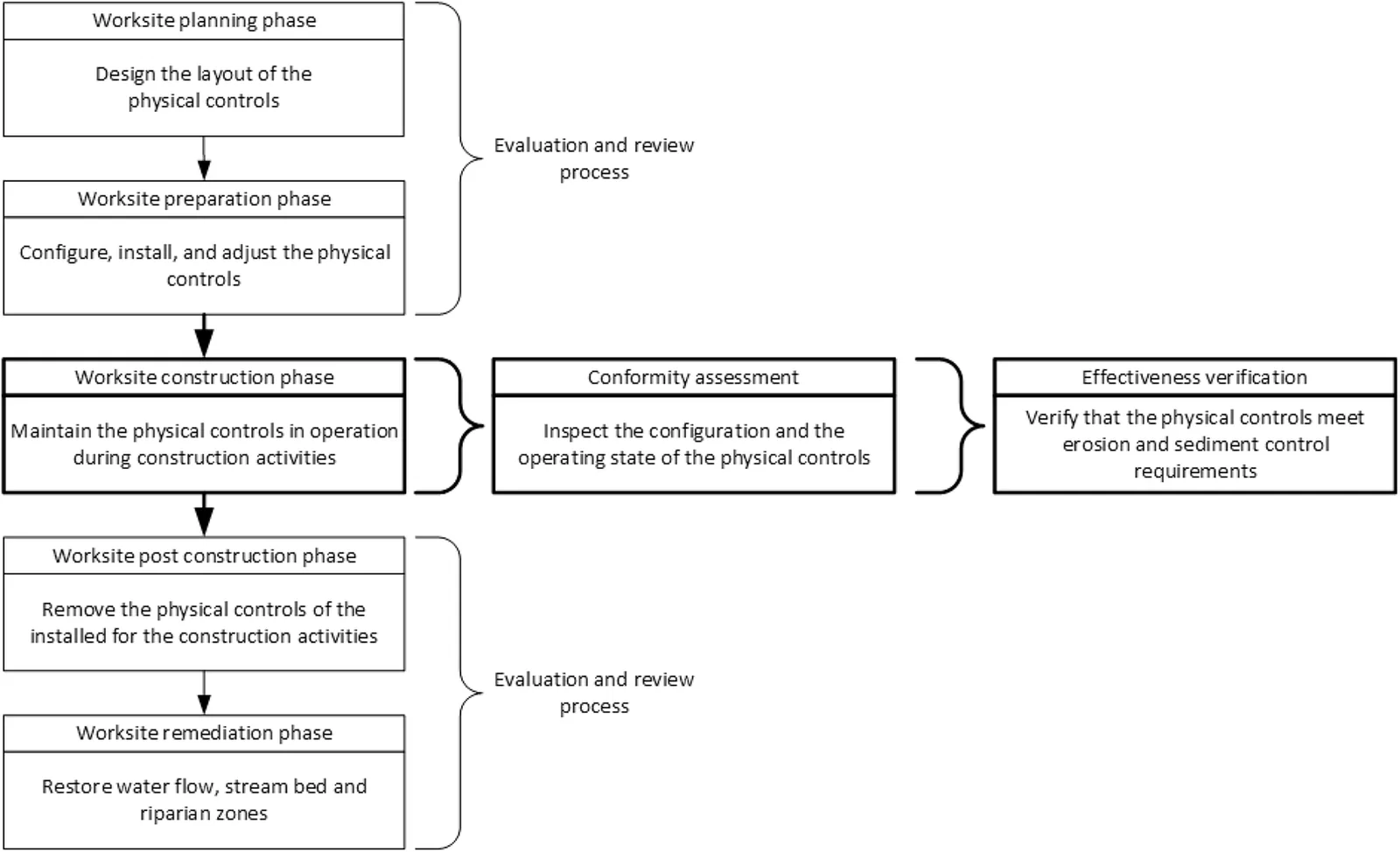
Worksite operational phases flowchart including conformity assessments and effectiveness verifications (bold framed boxes)

Using erosion and sediment control as an example, physical controls need to be maintained during the entire duration of the construction activities at a worksite (Fig. 3). The planning phase is used to design the layout of the physical controls to ensure that these can address the characteristics of the worksite and the expected environmental conditions that are likely to occur during the construction phase. It is during the worksite preparation phase that any adjustments are made while the installation of the physical controls is being finalized. Once the construction is completed, the physical controls are removed during the post construction phase. Restoration of a watercourse and riparian zones are some of the many activities undertaken during the remediation phase.

During the construction phase, there may be several conformity assessments made of the operating state of the physical controls. Conformity assessment is used to determine that the procedures, tasks, and maintenance requirements outlined in standards and codes of practice are being followed when they are used in the daily operation of a worksite (International Organization for Standardization 2006). When findings show that there are issues with the configuration or the maintenance and repair of physical controls, corrective actions are taken to address these. As part of an onsite inspection, a separate effectiveness verification is most useful when undertaken to verify that the configuration and installation of the physical controls are actually meeting the erosion and sediment control management benchmark at the time of the inspection to confirm the findings of a conformity assessment.

During the replacement and construction activities for both stream crossing worksites (Goguen et al. 2025), erosion and sediment control required the configuration of several physical controls including, sandbag cofferdams, lined bypass channels, mulching, silt fences and silt curtains, and many others. As part of the preparation of the worksite for the removal and replacement of the stream crossings, the configuration and installation of these controls had to allow for the worksite access of equipment and machinery, the use of a bypass channel of the stream, and the dewatering of the work area, for example. Once the construction activities were completed, the flow was returned to the natural watercourse, the physical controls were removed, and the riparian zones were restored as part of the planned remediation phase which is essentially the conclusion of the culvert replacement projects.

The results of the reliability analysis from Goguen et al. (2025) are used to demonstrate the development of an effectiveness verification sampling plan that could be used for other similar construction worksites. The reliability analysis used automated data logging to record turbidity data for 99 days at the Queens brook construction worksite in 2021 and for 110 days at the Kerr Brook construction worksite in 2022. The studies generated 4331 and 14,589 data points of turbidity respectively for each reliability period. In contrast to the reliability analysis that required extensive data during the entire construction phase to develop a standard or a code of practice, effectiveness verification is based on fewer observations because it is used to conclude that the requirement is met during the inspection at the worksite for a given day.

The protocols of the reliability analysis are available in Goguen et al. (2025). In summary, data loggers with turbidity sensors were installed approximately 150 m upstream of the worksite and approximately 60 m downstream of the worksite. Turbidity sensors were placed approximately 3 to 5 cm above the streambed to accommodate low summer water levels. Site visits were used to determine if the physical controls were functioning as intended (e.g. stable period) after configuration and installation (e.g. early period) or were being repaired because of wear and tear (e.g. wear-out period) or damage from stochastic environmental events that were outside the stated operating conditions (e.g. catastrophic periods). Within the context of a stated operating condition, water level fluctuations during rainfall events provided insight into the environmental conditions that could be considered beyond the design of the physical controls that would be established for a planning phase.

Based on Box 1, the performance of erosion and sediment control should not exceed a maximum increase of 8 NTU from background levels for short-term exposure. Here, the upstream NTU level represents the background level of turbidity as the water quality of the stream. The downstream level represents the residual turbidity generated from the physical controls during the construction phase. Thus, increases in NTU generated by the worksite activities are based on the difference between upstream and downstream NTU for each measurement *(*∆*Tu*). It is assumed that the turbidity level remains unchanged between the upstream sensor and the worksite sensor. As such, increases in turbidity observed by the downstream sensor are assumed to be generated from the worksite. It should be noted that this guideline was never exceeded during the construction phases of both projects. Although 8 NTU was exceeded during the worksite activities, the increase generally lasted for one to two hours of continuous turbidity measurements during the reliability study, hence meeting the less than 24 h of the guideline (Box 1).

Based on Table 1, the levels of non-conformity *(*∆*Tu* > 8 NTU) for each reliability period is based on the data extracted from the reliability analysis of Goguen et al. (2025). The variability in the levels of non-conformities across the two projects reflects the differences in the watershed characteristics as well as the stochastic environmental events that occurred during the construction phases. As per expectations for individual reliability periods of the Bathtub Curve, the weighted average of non-conformities show that the level for the early period (0.11) is higher than the stable periods (0.06) and increases sharply for the wear-out periods (0.60) and catastrophic periods (0.87). However, the individual reliability periods also show a broader range of non-conformity levels for each period.

**Table 1:** Level of non-conformity for ∆*Tu* > 8 NTU for individual and combined reliability periods (Fig. 2) of the two worksites during the construction phase

	Non-conformity levels by reliability periods			
Worksite location	Early	Stable	Wear-out	Catastrophic
Queens Brook (2021) Individual periods	-^a^	0.01	0.18	0.65
	-	0.18	0.03	0.73
	-	0.14	-	-
	-	0.003	-	-
Kerr Brook (2022) Individual periods	0.11	0.11	0.05	0.62
	-	0.04	0.74	0.97
	-	0.02	0.63	0.94
	-	-	-	1.00
Weighted averages for non-conformity levels	0.11	0.06^b^(SPQL)	0.60	0.87

^a^Data are not available for the early period for Queens brook because work started before the turbidity monitoring instruments were installed. ^b^System performance quality level (SPQL) derived from the stable period only.

The weighted average of the non-conformity level of the stable period of the reliability study is used as the system performance quality level to develop the acceptance sampling plan. The variability of the other reliability periods is also used to inform the selection of acceptable and rejectable quality levels.

## Acceptance Sampling Plan

The development of an acceptance sampling plan requires a policy that specifies the proponent’s risk, the environment’s risk, and the acceptable and rejectable quality levels in order to calculate the decisions rules for a given sample size. We use hypothetical policies to demonstrate the development of an acceptance sampling plan. In an environmental management context, an acceptance sampling plan is the “weakest link” given that a sample cannot be repeated once taken because of constantly changing reference conditions (Wagner 1995). In addition, a sufficiently large sample size is required to provide the statistical power to correctly detect whether technical measures are or are not effectively working as expected (Rumps et al. 2007; Pendrill 2014). Statistical power analysis is needed for the design of effectiveness verification sampling plans (based on reliability studies) to address Type I and Type II errors (Pertman 1990; Fairweather 1991; Taheriazad et al. 2023; Valicharla et al. 2024).

## Proponent’s Risk and Environment’s Risk

Although the mathematical probabilities methods used for acceptance sampling plans may be perceived as a mechanical process that makes decisions, a comprehensive effectiveness verification sampling plan is considered in terms of the policies that establish the level of protection being sought, the costs associated with such inspections, and the actions to be taken when non-conformities are found (International Organization for Standardization 2017; Suter 2019). Referring to the level of protection being sought, the probability of concluding that technical measures are in conformity does not only depend on the metrics being measured. It also depends on the probability that a given sample size, i.e. number of observations, can correctly detect the non-conformities during an inspection at the worksite.

Based on ISO 2859, an acceptance sampling plan should conclude that a producer is meeting a policy requirement when the true non-conformities respect the decision rule used to protect the consumer from such non-conformities. Given the environmental context of technical measures compared to manufacturing, we substitute the producer and the consumer for the proponent and the environment. The proponent is the person responsible for implementing and maintaining the technical measures during construction activities. The environment is what needs to be protected by an environmental policy. Type I Error is used as the proponents’ risk of concluding that a requirement is not being met when in fact it is (Box 2). In such a situation, the proponent may be penalized in error resulting in construction delays and accrued expenses. Type II Error is used as the environment’s risk of concluding that the conformity level is meeting a requirement when it is not. Thus, the environment may be inadvertently affected.

Therefore, the probability α for Type I Error is a measure of the proponent’s risk of concluding that a system of controls is not effective when it is. (Table 2). The probability β for Type II Error is a measure of the environment’s risk of concluding that a system of controls is effective when it is not. Inversely, 1-α is the probability of the correct decision for the proponent while 1-β is the probability of making the correct decision for the environment (Taheriazad et al. 2023). The probability 1-β is also an indicator of the Power of the test for the sampling plan to detect the occurrence of non-conformities needed to make the correct decision for the environment (Pertman 1990; Columb and Atkinson 2016; Uttley 2019; Wu et al. 2022).

**Table 2:** Type I and Type II errors and correct decisions related to four possible outcomes from effectiveness verification of a system of controls at a worksite

Acceptance sampling by attribute and decision rule	The system of controls meets the expected outcome	The system of controls does not meet the expected outcome
**Conclude that the system of controls is effective**	Correct (1-α) Correct decision for the proponent	Type II Error (β) Incorrect decision for the environment (Environment’s risk)
**Conclude that the system of controls is not effective**	Type I Error (α) Incorrect decision for the proponent (Proponent’s risk)	Correct (1-β) (Power of the test) Correct decision for the environment

An ideal acceptance sampling plan is expected to have low probabilities for α and β, implying a higher likelihood of reaching the correct decision for both the proponent and the environment. The development of such a sampling plan starts with the selection of α and β as risk-based policies in consideration of the proponent risk management requirements and the environmental protection policies being sought. It should be noted that the probabilities for α and β are different calculations and are not simply the inverse of the other (Pertman 1990).

In addition to a policy for the proponent’s risk (α) and environment’s risk (β) (Table 2), an acceptance sampling plan indexed by acceptable quality level (AQL) also needs a policy for acceptable and rejectable levels of non-conformities observed during a verification. The AQL is the maximum level of non-conformities that is considered satisfactory as a process average (Box 3). Considering the weighted average for the system performance quality level (SPQL) as equivalent to a satisfactory process average, the effectiveness of technical measures would be expected to have, on average, non-conformity levels as good as the AQL. Here, we also use the rejectable quality level (RQL) as the highest level of non-conformity above which a system of controls is deemed not effective. RQL is used to limit the level of non-conformities to address the environment’s risk.

Box 2 Proponent’s Risk and Environment’s Risk

- The **Proponent’s risk** is the probability of concluding that a system of controls is **not effective** when in fact it is **effective**. In acceptance sampling, this probability is identified by α.
- The **Environment’s risk** is the probability of concluding that a system of controls is **effective** when in fact it is **not effective**. In acceptance sampling, this probability is identified by β.

Box 3 AQL, RQL, and SPQL Definitions for Non-conformities of a System of Controls (Adapted from ISO 2859)

- **Acceptable quality level (AQL)** is the maximum level of non-conformity for which a system of controls is deemed effective.
- **Rejectable quality level (RQL)** is the highest level of non-conformity above which a system of controls is deemed not effective.
- **System performance quality level (SPQL)** is the weighted average of all non-conformity levels for a system of controls when it is performing as intended as defined by the stable periods of the Bathtub Curve.

## Single Sampling Plan

An acceptance sampling plan includes a sample size (i.e. number of observations per inspection) and decision rules that are based on the policies established for α and β and non-conformity levels for AQL and RQL (International Organization for Standardization 2017). The decision rule uses an acceptance number (*Ac*) for the non-conformities observed in a sample. The *Ac* is calculated for a given sample size of *n* observations, and the policies established for the proponent’s risk (α) and the AQL (1). If the number of non-conformities observed in the sample is less than or equal to the *Ac*, the verification will conclude that the system of controls is effective. When the number of non-conformities is greater than the *Ac*, the verification will conclude that the system is not effective.

Find the value of *Ac* such that

1 $$1-{\sum }_{d=o}^{{Ac}}\left(\begin{array}{l}{n}\\ {d}\end{array}\right){{AQL}}^{d}{(1-{AQL})}^{n-d}\le \,\alpha$$

In MS Excel

*Ac* = BINOM.INV(*n*,AQL,1-α)

*Ac *Acceptance number

*n* Sample size

AQL Acceptable quality level established as policy

α Proponent’s risk established as policy

## Operating Characteristic Curve

Whenever an acceptance sampling plan is used to verify the effectiveness of a system of controls, the true non-conformity level is not known. The probability of acceptance (*P*_*n,Ac,p*_), defined as the probability of observing *Ac* non-conformities or less in a sample of size *n* used during an inspection, is calculated for a given non-conformity level *p*, for 0 < *p* < 1, using the cumulative Binomial distribution (2).

2 $${P}_{n,{Ac},p}={\sum }_{d=0}^{{Ac}}\frac{n!}{d!\left(n-d\right)!}{p}^{d}{\left(1-p\right)}^{n-d}$$

In MS Excel

*P*_*n,Ac,p*_ = BINOM(*Ac,n,p*,TRUE)

*P*_*n,Ac,p*_ Probability of acceptance for non-conformity *p* less than or equal to the AQL for sample size *n*

*n* Sample size

*Ac* Acceptance number based on the AQL, and α of the policy

*p* Potential non-conformity level

**Fig. 4:**
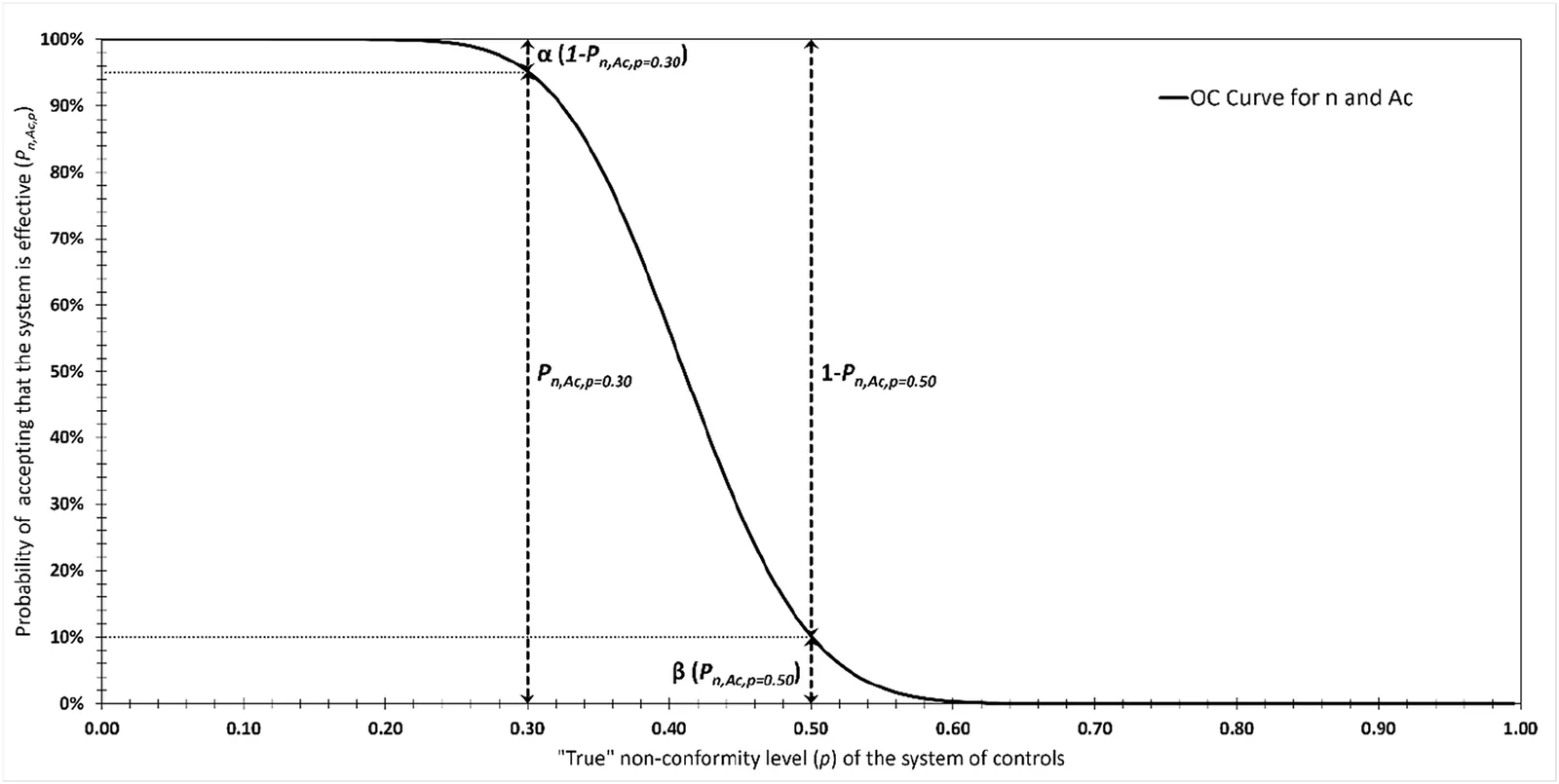
Example of an operating characteristic curve for sample size *n* and acceptable number *Ac*

For a set sample size *n* and acceptance number *Ac*, an operating characteristic curve (OC Curve) provides a visual representation of the probability of acceptance for the non-conformity levels for 0 < *p* < 1 (Fig. 4). The probability *P*_*n,Ac,p*_ decreases as the non-conformity level increases. In the example below, if a system of controls has a non-conformity level *p* of 0.30, there is a 95% probability for the effectiveness verifications (*P*_*n,Ac,0.30*_ = 95%) to conclude that the system of controls is effective using a sampling size of *n* and an acceptance number *Ac*. However, when non-conformity levels are 0.50, the probability for the effectiveness verification drops to 10% (*P*_*n,Ac,0.50*_ = 10%) of concluding that the system of controls is effective for the same sample size and *Ac*. This means that the power to detect a non-conformity level of 0.50 is 90% (*1-P*_*n,Ac,0.50*_). In absence of policy benchmarks for α and AQL as well as the β and RQL, an OC curve does not reflect the boundaries of acceptable or rejectable levels of non-conformity from a policy perspective.

### Development of an Acceptance Sampling Plan with Application of the Case Study

The acceptable quality level (AQL) is informed by the system performance quality level (SPQL) obtained from the results of the reliability study of erosion and sediment control (Table 2). Once the policy for acceptance sampling is established, the policy values for α and AQL are used to calculate the acceptance number (*Ac*) for the sample sizes being considered to verify that the level of non-conformity below which a system of controls is deemed effective. The values of β and RQL are used to determine the sample size with adequate power to detect the level of non-conformity above which a system of controls is deemed not effective. To demonstrate the development of a sampling plan, we use a hypothetical policy framework using an AQL (0.10) and an RQL (0.20) that are above the SPQL (0.06) in consideration of the risk to the proponent (α = 5%) and the environment (β = 10%) (Table 3).

**Table 3:** Example of hypothetical policies for an acceptance sampling by attribute

Policy (hypothetical) for acceptance sampling by attributes for a system of controls		
α	The Proponent’s risk is the probability of erroneously concluding that a system of controls is not effective when it is in fact effective.	5%
AQL	Acceptable quality level (AQL) is the maximum level of non-conformity for which a system of controls is deemed effective.	0.10
SPQL	System performance quality level (SPQL) is the weighted average of all non-conformity levels for a system of controls when it is performing as intended during the stable periods	0.06
β	The Environment’s risk is the probability of erroneously concluding that a system of controls is effective when it is in fact not effective.	10%
RQL	Rejectable quality level (RQL) is the highest level of non-conformity above which a system of controls is deemed not effective.	0.20

**Fig. 5:**
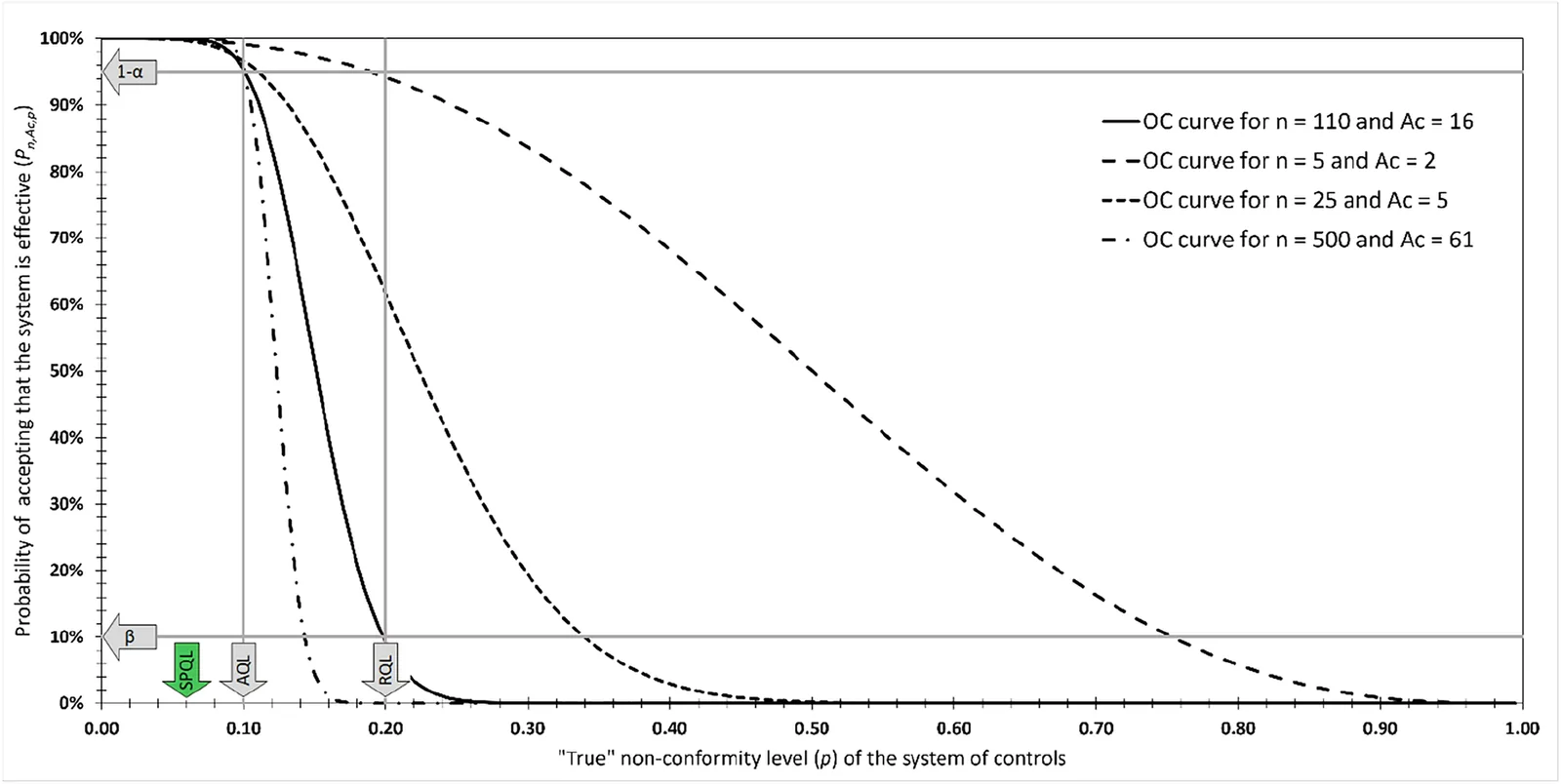
Operating characteristic curve for different sample sizes and acceptance numbers, overlayed with the policy benchmarks AQL and RQL for a correct decision for the proponent (1-α) and environment’s risk (β) policies

Applying the policy values of α and AQL (Table 3), the *Ac* (1) and probability *P*_*n,Ac,RQL*_ (2) are calculated for sample sizes used during an inspection of 5, 25, 110, and 500 (Table 4). Subsequently, the probabilities *P*_*n,Ac,p*_ for 0 < *p* < 1 are plotted as OC Curves (Fig. 5) (2).

**Table 4:** Sample sizes being considered for an acceptance attribute sampling plans for onsite inspections

Variables	Definitions	Sampling plans			
		*n* = 5	*n* = 25	*n* = 110	*n* = 500
*Ac*	**Acceptance number**: A system of controls is deemed effective when the number of non-conformities observed in the sample is less than or equal to the *Ac*	2	5	16	61
Type I Error (*1-P*_*n,Ac,AQL=0.1*_)	The probability of concluding that a system of controls is **not effective** when in fact it is effective	1%	3%	5%	5%
Type II Error (*P*_*n,Ac,RQL=0.2*_)	The probability of concluding that a system of controls is **effective** when in fact the level of non-conformities exceeds the RQL	94%	62%	9%	0.0003%
Power (*1-P*_*n,Ac,RQL=0.2*_)	The probability of concluding that a system of controls is **not effective** when in fact the level of non-conformities exceeds the RQL	6%	38%	91%	99.9997%

The OC curves (Fig. 5) is a valuable visual representation of the policy implications for the 4 sample sizes (Table 4) being considered for the development of a sampling protocol for an effectiveness verification. In consideration of the acceptance sampling policy, for a sample size to be considered adequate its OC Curve would have to pass above the intersection of the AQL (0.10) and 1-α (95%), and below the intersection of the RQL (0.20) and β (10%). By the mathematical selection of the *Ac* (1), for all sample sizes considered the probability *P*_*n,Ac,AQL*_ will be greater or equal to 95% (1-α), implying that the risk to the proponent, Type I Error (α), is always less or equal to 5%. The policy establishes tolerance levels for making an incorrect decision for the environment at a probability of 10% Type II Error (β), meaning that *P*_*n,Ac,RQL*_ should be less than 10%. Consequently, the sample size should have the power to detect non-conformities above the RQL with a probability greater than 90% (*1-P*_*n,Ac,RQL*_).

For the sample sizes considered during an inspection, the calculated probabilities *P*_*n,Ac,RQL*_ of the incorrect decision for the environment are 94%, 62%, 9%, and 0.0003% (Table 4 and Fig. 5). Considering the hypothetical policy (Table 3), only the sample sizes of 110 and 500 have a *P*_*n,Ac,RQL*_ lower than the policy of 10% (β). The OC Curves for the sampling plans of size 500 and 110 cross below the intersection RQL and β, thus would be adequate to address the environment’s risk (Fig. 5). In contrast, OC Curves for sample sizes 5 and 25 pass above the intersection of RQL and β, thus would not be adequate as they would have a *P*_*5,2,RQL*_ of 94% and *P*_*25,5,RQL*_ of 62%, which are greater than β.

The power of the sample can be used as a measure of the probability of concluding that a system of controls is not effective when in fact it is not when the non-conformity level is exceeding the RQL. Samples of 110 and 500 would have the power to detect if the non-conformity level is greater than the RQL with probabilities of *1-P*_*110,16,RQL*_ = 91% and *1-P*_*500,61,RQL*_ = 99.997%. However, sample sizes of 5 and 25 would only have the power to detect if the non-conformity level is greater than the RQL with probabilities of *1-P*_*5,2,RQL*_ = 6% and *1-P*_*25,5,RQL*_ = 38%. Smaller sample sizes have a lower power to detect non-conformities above the RQL resulting in a higher risk to the environment. Considering a policy tolerance of 10% (β), sample sizes of 5 and 25 would only detect at the power sought by the policy, at best, levels of non-conformities of 0.76 and 0.34 respectively which are greater than the RQL of 0.20 (Fig. 5).

Given the hypothetical policy for the RQL (Table 3), a sample size of 110 or greater should be selected for a sampling plan to have a P_*n,Ac,RQL*_ lower than the environment’s risk of 10% (β). Here, the sample sizes of 5 and 25 would be insufficient, i.e. *P*_*n,Ac,RQL*_ > 10%, and a sample size of 500 used for an inspection could be considered too stringent for the RQL policy (*P*_*n,Ac,RQL*_ ≪ 10%) when considering human and financial implications unless continuous data loggers are used to reduce the human resources that would be needed to record the observations. Given the absence of human presence when using automated data loggers, additional parameters should be monitored to confirm that the sampling occurred within the stated operating conditions. Using the OC Curve (Fig. 5), it is also clear that a reduction in β, for instance to 5%, would lower the intersection point of RQL and β and consequently require a larger sample size for its OC Curve to pass below the intersection.

**Fig. 6:**
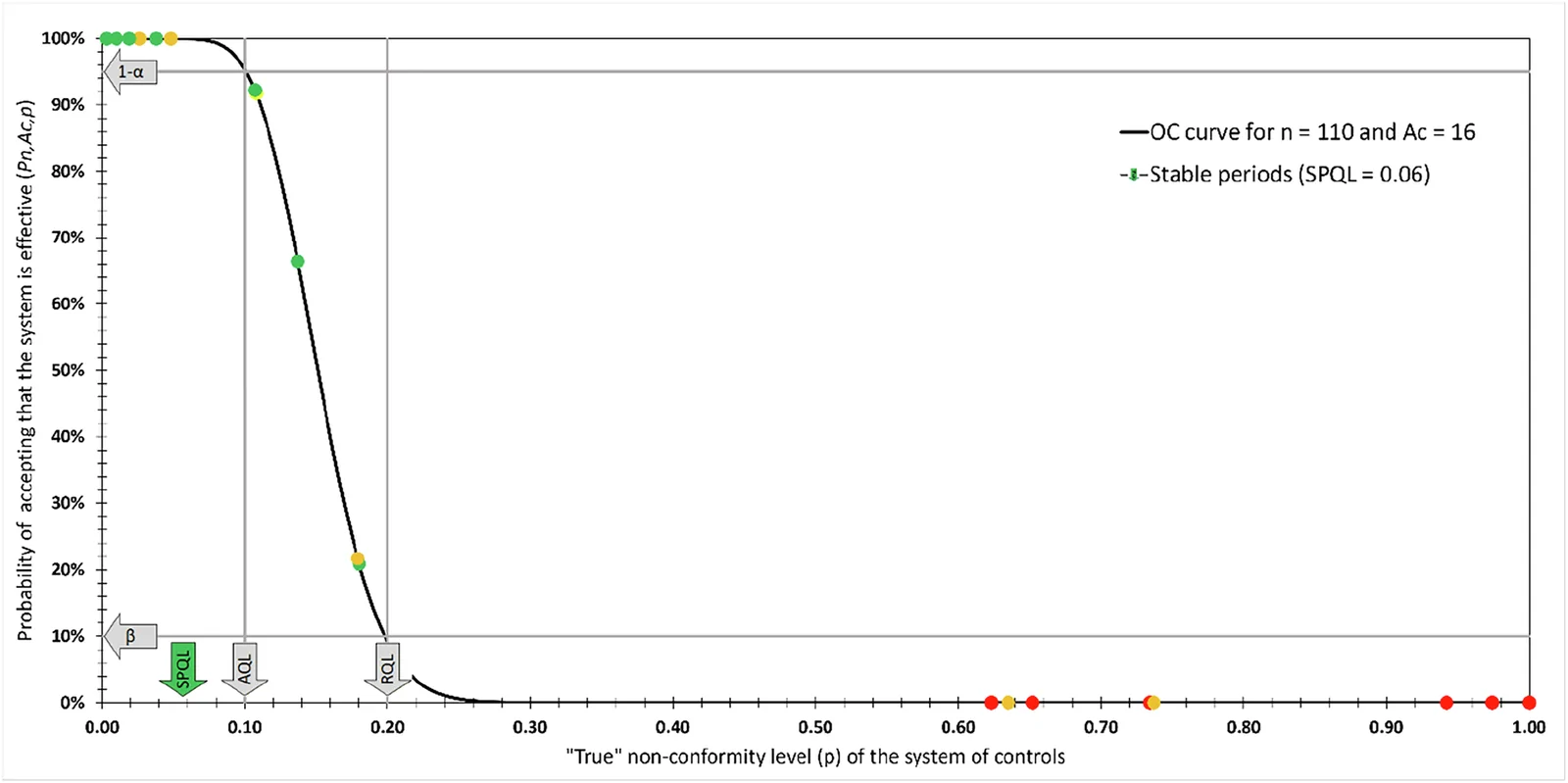
Operating characteristic curve for a sample size of 110, with an acceptable number of 16, and individual and combined non-conformity levels observed during the four reliability periods of the two case studies (stable in green, early in yellow, wear-out in orange and catastrophic in red), overlay with the SPQL from reliability studies and the policy set AQL, RQL, α and β

Working from a hypothetical effectiveness verification protocol for erosion and sediment control, the OC Curve is used to further demonstrate how the variability of the non-conformity levels for each individual reliability period can provide additional information to the SPQL (Table 1) used in the selection on the AQL. To fully understand the mechanics of a sampling plan decision, let us assume effectiveness verifications of erosion and sediment control are carried out within the context of the hypothetical policy (Table 3). It is shown above that the appropriate sampling plan should be based on a sample size of 110 for each future inspection. Figure 6 shows the OC Curve for this sampling plan and the observed non-conformity levels for the stable period (green points) of the two reliability studies. Non-conformity levels for early (yellow), wear-out (orange) and catastrophic (red) periods are also included to show the actual full range of observed non-conformity levels during the reliability study using continuous data logger recordings for the entire duration of the construction activities. Even though the SPQL is 0.06, the non-conformity levels for the individual stable periods range from 0.003 to 0.18. Variations in non-conformity levels are expected due to the inherent stochastic variations of a natural environment. An AQL set at, or slightly above, the SPQL, and an RQL set above the SPQL and AQL allow to account for the natural variability in the non-conformity levels. The non-conformity levels during the various stable periods are represented by green points on the OC Curve, and those points are mostly above the intersection of AQL and 1-α. For all periods with non-conformity levels that are less than the AQL, if a sample of 110 observations had been taken, the probability of concluding that the system is meeting the AQL policy benchmark would have been well over 95%. For the three green points where non-conformity level during the period exceeded the AQL, if a sample of 110 observations had been taken during those periods the probability of concluding that the non-conformity levels are below the AQL would have been *P*_*110,16,0.11*_ of 92%, *P*_*110,16,0.14*_ of 67% and *P*_*110,16,0.18*_ of 22% (Fig. 6). This means that if a sample of 110 observations is taken there is a 22% probability of observing 16 or less non-conformities when the true non-conformity level is 0.18, which is above the AQL (0.10). When using a sample size that adequately address the policy for AQL and RQL, the probability of concluding that the system is effective when its non-conformity level is greater than AQL will rapidly decrease as the non-conformity levels approach the RQL and drops below 10% when the true non-conformity level is greater than the RQL.

**Fig. 7:**
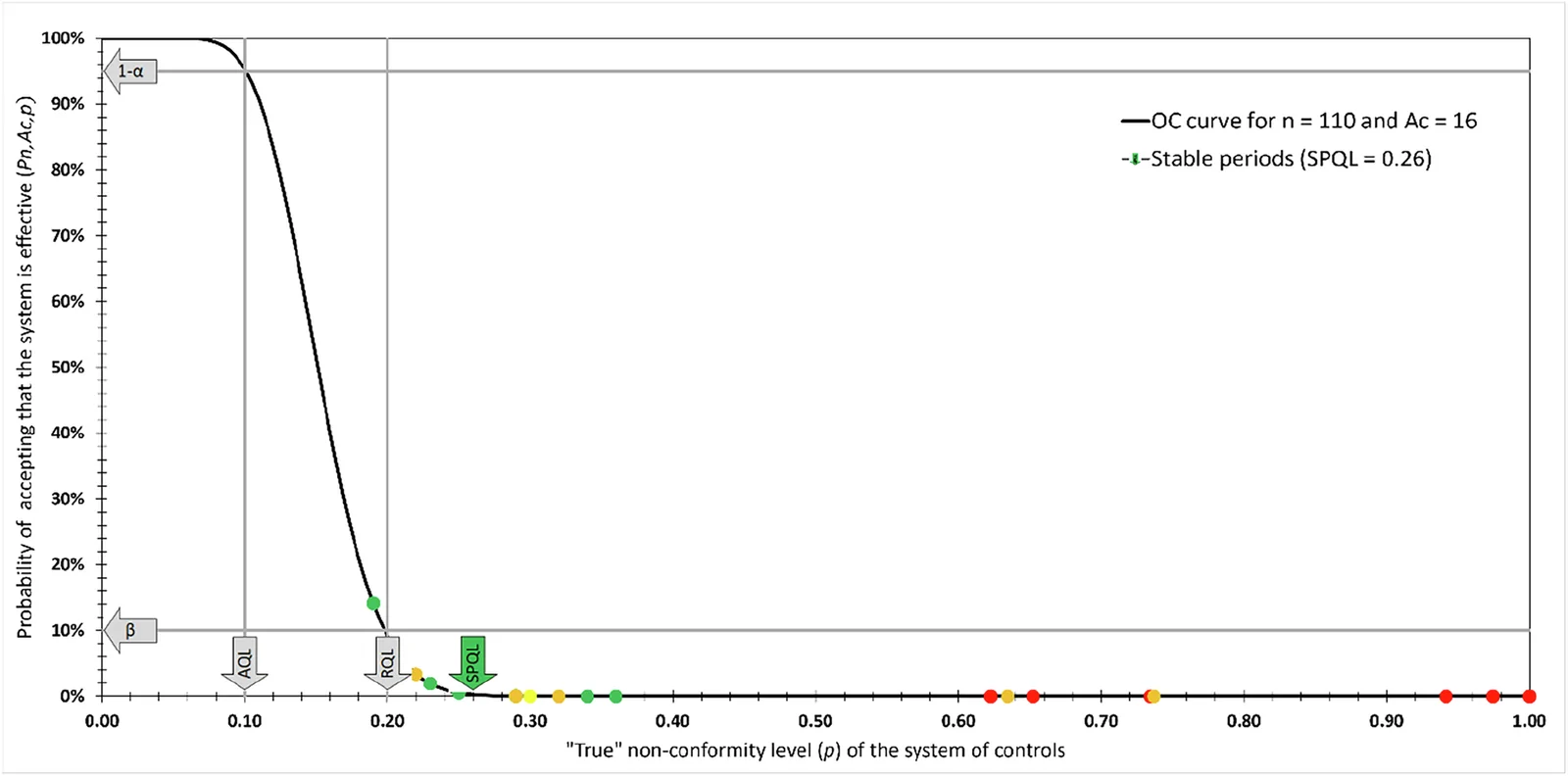
Operating characteristic curve for a sample size of 110, with an acceptance number of 16, and individual and combined hypothetical non-conformity levels for the four reliability periods for demonstration purposes (stable in green, early in yellow, wear-out in orange and catastrophic in red), when the AQL and RQL are not determined based on a reliability study and are below the average non-conformity level SPQL

A sampling plan used for effectiveness verification mechanically concludes that the system is effective or not effective regardless of a policy for AQL and RQL. However, the conclusions may not be in line with the SPQL if the AQL and RQL are set lower than the SPQL that can be achieved by the standard or the code of practice used for a system of controls. Therefore, if the AQL is set lower than the SPQL, most of the effectiveness verifications using this sampling plan would erroneously conclude that the system is not effective even though the system is adequately performing its intended function given the SPQL level (Fig. 7). Assuming that SPQL is 0.26 and the AQL and RQL are set at 0.10 and 0.20, the OC Curve highlights that the probability of acceptance P_110,16,SPQL_ would only be 0.3%. A sampling plan with an AQL and RQL that are lower than the SPQL would result in an increase in the proponent’s risk because of a very low rate of acceptance even though the implementation of the system of controls meets the requirements of a standard or a code of practice when implemented at a worksite. This would be an indication that higher AQL and RQL would be needed to adequately address the SPQL because it is considered as the indicator for a reliable system of controls.

## Discussion

Effectiveness verification is used for real time onsite inspections in the field, and not to assess an impact after the construction activities are completed at a worksite. The purpose of such verifications is to determine if the approved technical measures are meeting the requirements of regulations, standards, and codes of practice that are used to protect the environment during the construction activities at a worksite. Effectiveness verification is used in tandem with conformity assessments to ensure that issues found with the system of controls are addressed by corrective actions at the time of the inspection and not after the construction is completed. Acceptance sampling needs decision rules that are based on an acceptable quality level (AQL), a rejectable quality level (RQL), and informed by a system performance quality level (SPQL) that is generated from prior reliability studies of higher detailed sampling of similar systems of controls to address the regulatory implications of the proponent’s risk and the environment’s risk.

The effectiveness verification sample size and decision rules can only detect if non-conformity levels meet an AQL or exceed the RQL regardless of the reliability period the verification takes place. Protocols for integrating effectiveness verification with conformity assessments would be needed to ensure that a verification is used to confirm the findings of a conformity assessment during the stable period. Conducting an effectiveness verification during the other reliability periods would only confirm that the installation of the physical controls is yet to be finalized during the early periods, that repairs are needed or ongoing during the wear-out period, or to address the damage from stochastic environmental events during the catastrophic periods. For these three periods, effectiveness verifications (collection of turbidity observations) would not necessarily add more information than what would have been confirmed via a conformity assessment. Without the knowledge of the results of an inspection or a conformity assessment, an effectiveness verification could be biased. For example, in cases where some physical controls are in disrepair, but are not causing increases in turbidity during low rainfall periods at the time of the verification, the effectiveness verification would conclude that the system of controls is effective even though a conformity assessment would have found issues with the physical controls.

In this paper, we demonstrate the development of an acceptance sampling plan that specifies the effectiveness verification sample size and decision rule based on acceptable and rejectable quality policies and risks of making an incorrect decision for the proponent and the environment. Such sampling plans could be used by a proponent of a project or by a person responsible for conducting independent verifications (e.g. competent authority or a certification authority). Here, we demonstrate the need for a conformity attribute and SPQL to develop an effectiveness verification sampling plan based on a policy for the proponent’s risk (α), the acceptable quality level (AQL), the environment’s risk (β), and the rejectable quality level (RQL). However, the use of such a sampling plan also requires a sampling protocol that would include specifications such as manual or automated data collection and the location and time frame that observations are to be taken. For example, a sampling protocol for erosion and sediment control would include specifications for upstream and downstream sampling locations in relation to the worksite, among other parameters. Such protocol would also specify that, for an inspection, the sample of *n* observations should be taken for over a minimum of 24 h given the turbidity benchmark of exceeding 8 NTU for short-term exposure of over 24 h (Box 1).

Reliability analysis is a well-established technique in engineering. It provides a structured framework to understand the performance of a system of controls such as the case here with erosion and sediment control. Such studies require extensive data collection and resources to determine the variability in the performance of such a system used at a worksite. Reliability analysis is best suited to inform the development of effectiveness verification acceptance sampling plans that are used to determine if the system of controls meet a conformity attribute at the time of the onsite inspection instead of measuring the performance of such system during the entire duration of worksite activities. Acceptance sample sizes and decision rules are used to make an immediate decision regarding conformity at the worksite. However, both reliability analysis and effectiveness verification need management benchmarks for environmental protection policies as provided by the CCME guidelines for total suspended sediments. Without such a benchmark, technical measures do not have any expected outcomes for a reliability analysis, and an inspection does not have the conformity attribute to make a correct decision during an effectiveness verification.

Even when a system of controls is performing its intended function, there will always be a probability of non-conformities occurring. Thus, non-conformity levels of any system of controls are never zero. This is the reason why acceptance sampling plans use an acceptance number for non-conformities as a decision rule for a given AQL that recognizes that non-conformities may occur when the system of controls is performing as intended. For example, a maximum increase (downstream of the worksite) of 8 NTU (∆*Tu*) from background levels (upstream) was exceeded during the reliability studies at both worksites. However, the increases lasted for short periods of time that were less than 24 h and, thus, never exceeded the CCME guidelines. It highlights the need for an onsite inspection sample with sufficient observations to make the correct decision for the proponent (Type I Error) and the environment (Type II Error). Acceptance sampling plans by attribute using Type II Error (β) for the environment’s risk quantitatively links the conclusion made during field verifications to the environmental protection policy objectives. Informed by reliability studies, it also incorporates stochastic variability of non-conformity levels into the development of such sampling plans. From an environmental protection perspective, a low β for Type II Error provides assurance that correct decisions are made when a system of control does not function as intended. In other words, for effectiveness verification an acceptance sampling plan with adequate sample size has the power to detect when conformity levels exceed the RQL.

## Conclusion

This paper demonstrates the use of acceptance sampling by attributes to verify the effectiveness of technical measures used as standards and codes of practice at construction worksites. An effectiveness verification sampling plan must be based on management benchmarks for the protection of the environment. Detailed studies such as reliability analysis provide valuable insight into the variability of non-conformity levels for a satisfactory implementation of technical measures for the development of such sampling plans. The sampling plan for effectiveness verification must also address a policy for the proponent’s risk and the environment’s risk. Benchmarks and acceptance sampling plan policies are required to establish decision rules to make a correct decision during an effectiveness verification.

The engineering techniques and international standards introduced in this paper have been used for decades. However, we are of the view that the applications of these techniques and standards provide a bridge between environmental protection policies and the implementation of technical measures in a regulatory context. It also provides clear expectations for the proponent who is accountable for the implementation of the technical measures. More importantly, the environment’s risk identified by the Type II Error provides a transparent metric for the expectations of environmental protection policies.

## Data Availability

No datasets were generated or analysed during the current study.
